# Sustainable dyeing of cotton, silk and leather using natural dye from *Bixa orellana* seeds: extraction, optimization and assessment of antibacterial activity

**DOI:** 10.3389/fchem.2025.1474160

**Published:** 2025-05-02

**Authors:** Moorthy Muruganandham, Yuvaraj Tamilselvi, Kanagasabapathy Sivasubramanian, Palanivel Velmurugan, Fatimah Oleyan Al-Otibi, Subpiramaniyam Sivakumar

**Affiliations:** ^1^ Centre for Materials Engineering and Regenerative Medicine, Bharath Institute of Higher Education and Research, Chennai, Tamil Nadu, India; ^2^ Department of Botany and Microbiology, College of Science, King Saud University, Riyadh, Saudi Arabia; ^3^ Department of Bioenvironmental Energy, College of Natural Resources and Life Science, Pusan National University, Busan, Republic of Korea

**Keywords:** ultrasound, extraction, *B. Orellana*, FT-IR, UV-spectrophotometer, bacterial reduction

## Abstract

**Introduction:**

Natural dyes offer an eco-friendly alternative to synthetic colorants in textile processing. This study explores the sustainable dyeing of cotton, silk, and leather using pigment-rich extracts from *Bixa orellana* seeds, with a focus on process optimization and antibacterial efficacy.

**Materials and methods:**

Using an array of extraction solvents [water, methanol, ethanol, ethanol: methanol (1:1)] and extraction techniques [direct heat (DH), ultrasonic water bath (USB), ultrasonic probe, (USP)], *Bixa orellana* seeds were utilized to produce a yellow-orange dye. The color strength of the extracted dye was investigated using a UV-Visible spectrophotometer to measure the absorbance wavelength. The functional groups identified in the extracted natural dye were described using an FTIR spectrophotometer.

**Results and discussion:**

Using methanol as the solvent and 5 g of seed at 60°C for 60 min, the highest color yield was observed in USB. Using the ultrasonic water bath dyeing method at 60°C for 40 min without using any moderant, cotton, silk fabric, and leather were effectively colored in a yellow-orange color. The L*, a*, and b* values of the dyed material treated using the USB approach were 80.95, 4.52, 75.35 for cotton, 88.65, -1.35, 62.85 for silk, and 79.55, 015.35, 66.45 for leather, respectively. Compared to the other bacterial reduction, 85.25% of the colored materials showed substantial antibacterial action against *Staphylococcus* sp. *Vibrio* sp. (76.69%), *Pseudomonas* sp. (75.83%), *Klebsiella* sp. (74.24%), and *Micrococcus* sp. (74.21%) were the following most prevalent bacteria. The ultraviolet protection factor (UPF) measurements showed that leather and cotton treated with *B. orellana* seed dye had higher UV radiation shielding properties.

## 1 Introduction

Dyeing is a process that involves coloring textile items to enhance their appearance, requiring colorants that can adhere to the fabric, whether they are natural or synthetic. Natural dyeing has a long history around the globe (Rather et al., 2020). However, the methods used might vary greatly depending on the availability of adjacent plants and minerals that can be used to make colors. Before the invention of artificial dyes in 1856, organic coloring ingredients were widely employed. However, when chemical dyes became more common, the usage of natural dyes decreased (Saxena and Raja, 2014). Unfortunately, prolonged use of chemical dyes started to have adverse health impacts, leading to skin disorders and other health problems from donning artificially colored garments (Tausif et al., 2018). Due to increased environmental, ecological, and pollution concerns, along with an improved comprehension of the health hazards and toxicity involved in their manufacturing, processing, and application, the textile industry is gradually reducing the utilization of synthetic dyes (Moore and Ausley, 2004). Natural dyes have become a significant replacement for artificial colors in recent years. Natural dyes can be used eco-friendly for dying fabrics because of their creative possibilities, good color fastness, repeatability, non-polluting nature, and soft, glossy, subtle, and bright colors. As natural colors become popular, researchers are looking into different plant components to find new sources of natural dyes. Natural dyes have a great aesthetic appeal, are durable, and have a varied spectrum of harmonic color combinations that generate gorgeous muted tones as they fade over time (Samanta et al., 2018).

Various plant sources contain natural colors, such as roots, seeds, leaves, fruits, and flowers. *Bixa orellana* L. is a member of the Bixaceae family and is commonly known as annatto. This plant grows up to 3–6 m tall and is one of the earliest known natural dye-producing plants. Its name was derived from the Spanish conquistador Francisco de Orellana, and it was formerly employed for body painting, relieving heartburn and stomach aches, sun protection, fending off insects, and warding off evil (Silva et al., 2010). Indigenous people have traditionally used the seeds of this plant to enhance lip beauty, which has resulted in the nickname “lipstick tree” for *Bixa orellana* (Aher et al., 2012). In the past few decades, several studies have shown the extraction of various classes of phytoconstituents from all parts of this plant, including carotenoids, apocarotenoids, sterols, aliphatic compounds, monoterpenes, sesquiterpenes, triterpenoids, volatile oils, and other unspecified compounds. These phytochemicals have diverse pharmacological effects, such as antibacterial, antifungal, antioxidant, anti-inflammatory, anticancer, improved gastrointestinal motility, neuropharmacological, anticonvulsant, analgesic, and antidiarrheal activity (Vilar et al., 2014).

While *B. orellana* has been the focus of numerous studies as a natural dye, our study provides an in-depth look at optimizing the dyeing process for three distinct substrates, cotton, silk, and leather, under various conditions. This is a novel approach to dyeing (Pizzicato et al., 2023; Rather et al., 2023). Sustainable Dye Extraction Techniques: Our study is on environmentally friendly and sustainable dye extraction techniques that reduce their adverse environmental effects. To demonstrate how our method offers the green chemistry perspective, we will provide a thorough comparison with current methods (Usman et al., 2023; Putra et al., 2023; Pranta and Rahaman, 2024). Antibacterial Activity Assessment: While *B. orellana* has been studied for its coloring properties, its antibacterial potential when applied to textile and leather materials has not been extensively reported. Our study evaluates this aspect, providing new insights into multifunctional applications of the dye (Medina-Flores et al., 2016; Dos Santos et al., 2022; Valarezo et al., 2023).

Natural dyes can be extracted from their sources by lowering resistant mass transfer through enhanced solvent sample contact and interactions (Esclapez et al., 2011). Various established and advanced technologies or techniques have been used to extract dye from natural sources. Conventional processes of extraction include distillation, solvent extraction, maceration, heat treatment, and soxhlet extraction, which have been used for a long time (Pandey and Tripathi, 2014). However, these procedures have drawbacks: they consume time, energy, and solvent, have low extraction yields, and cause the loss of thermolabile molecules.

Non-thermal or non-conventional technologies are being researched to alleviate the disadvantages of conventional methods (Poojary et al., 2016). Emerging technologies like supercritical fluid extraction, microwave-assisted extraction, ultrasound-assisted extraction (UAE), high-pressure homogenization, pulsed electric fields, high voltage electrical discharges, light stresses, and enzyme-assisted treatment have been proposed, developed, and improved. UAE is among the most recognized and accomplished new methods for recovering natural pigments (Wani et al., 2021; Sharma et al., 2021). According to Mason et al. (1996), the primary mechanism of ultrasonic extraction was the amplification of mass transfer *via* ultrasound and the fast access of solvent to plant cell components.

In the present study, the optimization of dye extraction from *B. orellana* seed was done using various methods, such as direct heat (DH), ultrasonic water bath (USB), and ultrasonicator probe (USP). Sustainable dyeing of cotton, silk, and leather using the extracted dye and optimizing the parameters of the dyeing process were also examined. Furthermore, dyed materials were evaluated for their color fastness and antibacterial properties.

## 2 Materials and methods

### 2.1 Extraction of natural dye

Dried seeds of *B. orellana* were purchased from the Merwar Impex store in India. The seeds were pulverized and sieved to get fine powder. *B. Orellana* natural dye extracted using water, ethanol, methanol, and ethanol: methanol (1:1 ratio) mixture in various extraction methods like DH, USB, and USP. Briefly, 5 g of powdered *B. Orellana* was weighed and mixed with 50 mL of each solvent in Erlenmeyer flasks for each method separately. DH method of extraction: The dye was extracted using the heating mantle as a heating source in assorted temperatures (30°C, 40°C, 50°C, 60°C, and 70°C) for 10 min. Similarly, the USB extraction of dye was carried out in an Ultrasonic water bath (LABMAN, LMUC-25, India) at various temperatures (40°C, 50°C, 60°C, 70°C and 80°C) maintained for 30 min and the USP method of extraction was assessed by Ultrasonic Probe Sonicator (LABMAN, Pro-650, India) with different power output (390, 455, 520, 585, and 650 W) for 30 min. The probe temperature was maintained at 50°C to avoid potential heat damage. The absorption spectra of extracted dye were measured using UV-1800, Genesys 180, and UV-Vis spectrophotometer (Thermo Fisher Scientific, United States). The dye extract with the maximum color strength was used to optimize the extraction levels of temperature, time, and substrate concentration (Velmurugan et al., 2016).

### 2.2 Optimization of parameters for dye extraction

Natural dye extraction from the powdered *B*. *orellana* seed was optimized using various parameters using USB with solvent as methanol. Different amounts of dye powder were at 10, 20, 30, 40 and 50 mg/L of solvent, and the temperature was controlled at 60°C for 60 min. Time 10, 20, 30, 40, 50, and 60 min and temperature 30°C 40°C, 50°C, 60°C, and 70°C. To minimize solvent loss and ensure reliable data, the extractions at 70°C were performed in a closed system, which reduced evaporation effects. Additionally, solvent volume was carefully monitored throughout the process. Optimization must be performed to extract dye at optimal concentrations. The absorbance of the extracted dye was measured using a UV-visible spectrophotometer in the wavelength range from 200 to 800 nm.

### 2.3 FTIR spectroscopy

The functional groups of the extracted dye were identified using FTIR spectroscopy. The spectrum was acquired using a Nicolet Summit LITE FTIR spectrometer from Thermo Fisher Scientific, and it was recognized from 4,000 to 500 cm^−1^ with a resolution of 4 cm^−1^. Five copies of each sample were scanned, and the average spectrum was smoothed and adjusted for baseline using the Spectrum for Windows software before the data were analyzed further (Sudharsan et al., 2016).

### 2.4 Dyeing

The dyeing efficiency of the extracted dye from the *B. orellana* seed was evaluated on cotton, silk fabrics and leather. Like extraction methods, the dyeing process was assessed using DH, USB, and USP methods. The cotton and silk fabrics purchased from local textile shops were meticulously cleaned and washed before dyeing. Likewise, the surface of the leather material was cleaned to get rid of the hair follicles and any other materials that adhere to the leather. All the dyeing processes were conducted with a constant 10% dye concentration. Optimization of the dyeing process was carried out at different temperature (40°C, 50°C, 60°C, 70°C and 80°C) with fixed time 50 min in an ultrasonic bath, various power output (390, 455, 520, 585, and 650 W) for 50 min in the ultrasonic probe and various temperature (30°C, 40°C, 50°C, 60°C, and 70°C) in heating mantle for 10 min, respectively. Each dyeing method was analyzed using 1″ × 1″square dyeing material (Cotton, Silk fabrics and leather). The dyed materials were subsequently rinsed in cold water and a 2 g/L concentration of nonionic detergent (laboratory detergent) (Velmurugan et al., 2016).

### 2.5 Ratio of liquor-to-fabric

The dye exhaustion in cotton, silk, and leather during the dyeing process was analyzed by following the previous method with minor alterations (Sivakumar et al., 2009). At consistent interludes, spent dye liquor samples were collected from each dye bath to analyze cotton, silk, and leather dye content. The % dye exhaustion was calculated using Equation 1.

$$\%\;dye\;exhaustion=\frac{Dye\;powder\left(g\right)-dye\;powder\;in\;spent\;liquor\left(g\right)}{Dye\;powder\left(g\right)}\times 100$$ (1)

### 2.6 Assessment of color characteristics

The color strength (K/S) and CIE L* a* b* coordinates of the dyed cotton, silk and leather were evaluated using Data Color 600 spectrophotometers from Data Color Company (United States) under illuminant D65 and a 10° standard observer. The Kubelka-Munk equation (Equation 2) was used to compute the color strength (K/S) in the visible part of the spectrum (400–700 nm).

$$K/S=\left(1-R\right)2/2R$$ (2)

Where K is the absorption coefficient, R is the reflectance of the dyed sample, and S is the scattering coefficient. The L*, a*, and b* hue spectrum statistically defines each perceptible color in three dimensions. L* represents brightness, and a* and b* represent the color constituents green-red and blue-yellow.

### 2.7 Determination of color fastness

Various color fastness tests were conducted on dyed cotton, silk, and leather. The rubbing fastness test of the dyed leather samples was examined according to ISO 11640:2012, the Color fastness to washing of the dyed silk sample was observed according to ISO 105-X12, and the light fastness test was evaluated according to ISO 105-B02 for dyed cotton fabric samples.

### 2.8 Antibacterial activity of dyed specimens

The antibacterial efficacy of dyed materials was evaluated using the quantitative AATCC 100 test procedure. Dyed and undyed materials (2 cm × 2 cm) were taken in a culture flask containing 50 mL of Mueller Hinton Broth. They were tested against skin pathogens (*Staphylococcus* sp., *Pseudomonas* sp., *Vibrio* sp., *Klebsiella* sp., and *Micrococcus* sp.). The flask was incubated at 37°C for 24 h in a shaker at 120 rpm. After incubation, 100 mL of distilled water was added to the flask and mixed well before making the three-time dilution of the supernatant. The diluted aliquots were plated on nutrient agar and incubated for 24 h at 37°C. Viable colonies of bacteria on the agar plate were counted, and the percentage of the reduction in the number of bacteria was calculated using Equation 3.

$$R\left(\%\right)=\left(A-B\right)/A\times 100$$ (3)

where (R) is the percentage reduction of bacteria, (A) represents the number of bacteria colonies in control (the untreated fabric), and (B) represents the number of bacteria colonies in the treated fabrics (Metwally et al., 2021).

### 2.9 Ultraviolet protection factor (UPF)

According to Pisitsak et al. (2016), Dry and wet cotton, silk and leather with appropriate controls were evaluated for their UV protection properties using a UV–vis–NIR spectrophotometer (Shimadzu (Japan) UV-NIR-3600) according to AATCC Test Method 183-2010. The ultraviolet protection factor (UPF) was calculated using the following Equation 4:

$$UPF=\frac{\sum_{280nm}^{400nm}Ex-Sx-\Delta \lambda }{\sum_{280nm}^{400nm}Ex-Sx-Tx-\Delta \lambda }$$ (4)

where *E*_x_ = relative erythemal spectral effectiveness. S_λ_ = solar spectral irradiance. Tx = average spectral transmittance of the specimen (measured). ∆λ = measured wavelength interval (nm).

The average transmittance values in the A- and B-UV regions, *T*(UV-A) _AV_ and *T*(UV-B) _AV_, were calculated using the following Equations 5, 6:

$$T\left(UV-A\right)A_{v}=\frac{\sum_{315nm}^{400nm}T\lambda \times \;\Delta \lambda }{\sum_{315nm}^{400nm}\Delta \lambda }$$ (5)

$$T\left(UV-B\right)A_{v}=\frac{\sum_{280nm}^{315nm}T\lambda \times \;\Delta \lambda }{\sum_{280nm}^{315nm}\Delta \lambda }$$ (6)

## 3 Results and discussion

**FIGURE 1:**
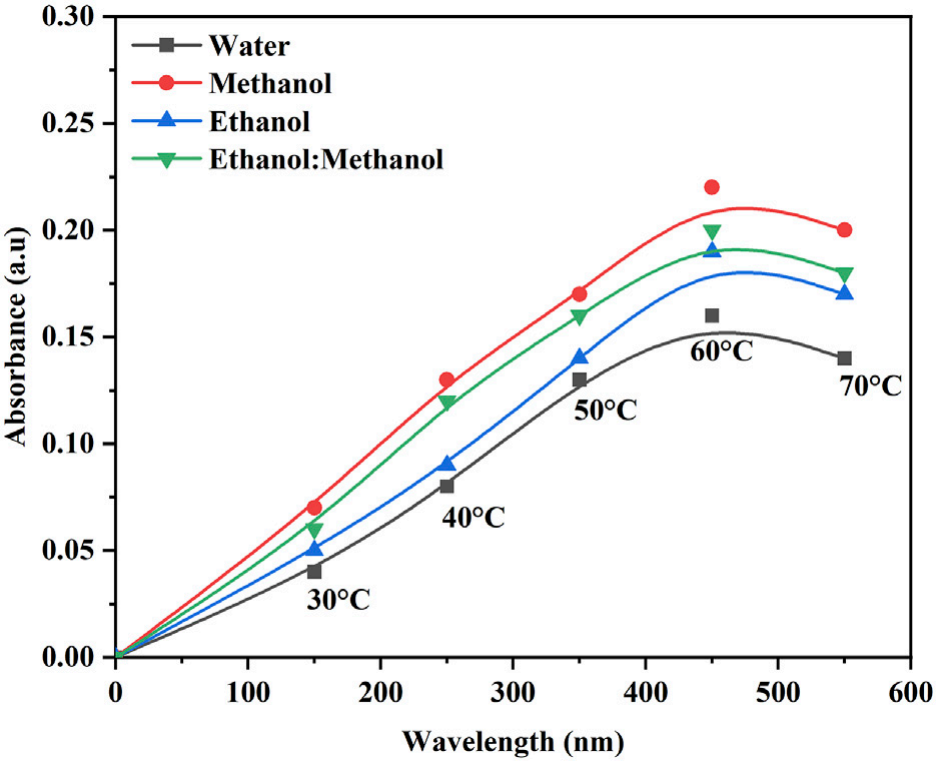
Optimization of *B. orellana* dye extraction using direct heat in different temperatures and solvents water, methanol, ethanol, and ethanol: methanol.

**FIGURE 2:**
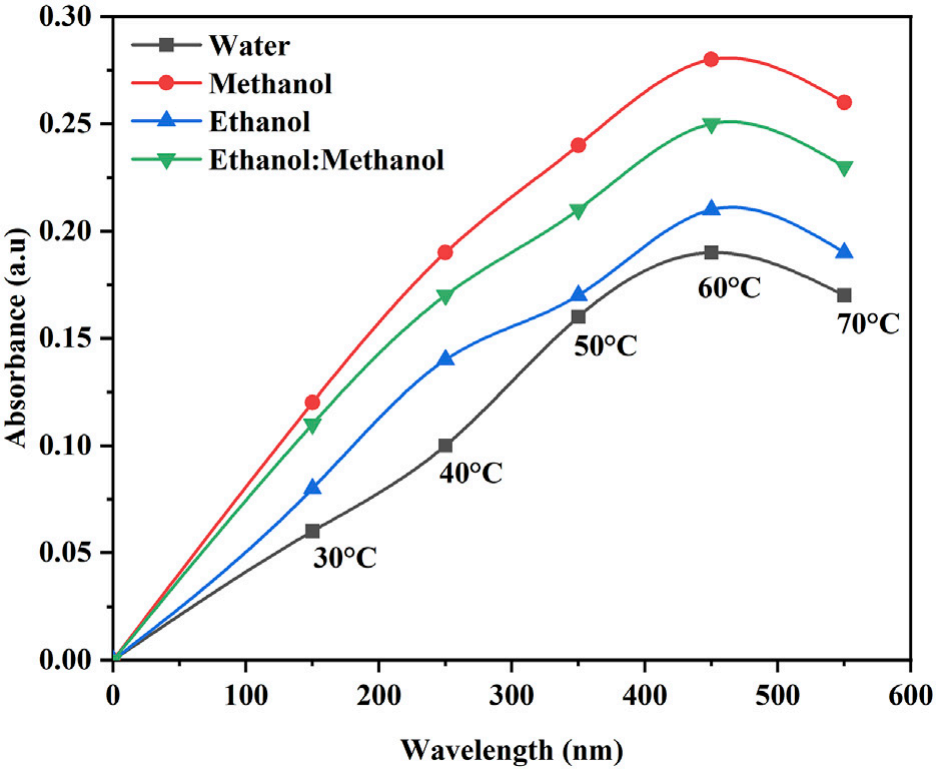
Optimization of *B. orellana* dye extraction using an ultrasonic water bath in different temperatures and solvents water, methanol, ethanol, and ethanol: methanol.

**FIGURE 3:**
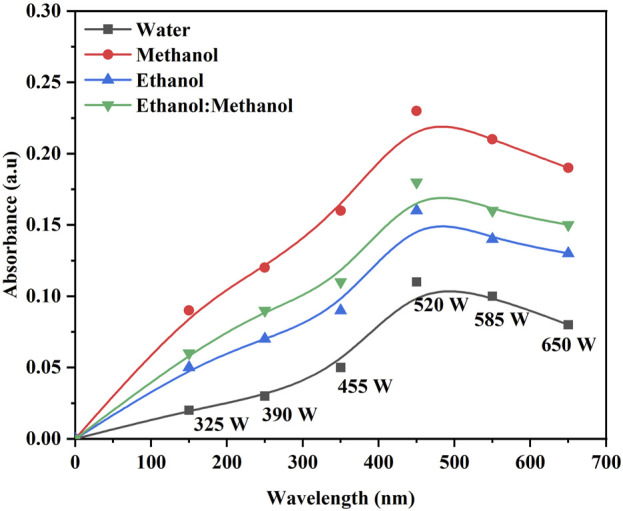
Optimization of *B. orellana* dye extraction using an ultrasonic probe in different output power (W) and solvents water, methanol, ethanol, and ethanol: methanol.

The color strength in the DH extraction method depends on the temperature and solvent used. The maximum color intensity was attained in the methanol extraction solvent followed by ethanol: methanol (1:1), ethanol and water (Figure 1). The UV-visible spectrum confirms the maximum color intensity observed in solvent methanol (Figure 1) at 60°C, followed by a mixture of ethanol: methanol, ethanol, and water. The dye yield was achieved at the same temperature. Color intensity was low in lower temperatures and high in higher temperatures, respectively. The color intensity of the extracted dye was obtained using the USB extraction technique when the extraction temperature was varied from 30°C to 70°C for 30 min. At 60°C, the maximum color intensity was observed (Figure 2). The lowest color intensity was observed in water and ethanol, followed by the highest in methanol and ethanol: methanol mixture. Research findings on dye extraction highlight the increased impact of power ultrasonic, which facilitates dye extraction from annatto *via* dispersion, degassing, and diffusion (Kamel et al., 2005). In the case of the USP-mediated extraction using varying power outputs (325, 390, 455, 520, 585, and 650 W) at a constant temperature of 50°C for 30 min, the maximum color intensity obtained at 520 W (Figure 3). The lowest color intensity was observed in water and ethanol, followed by the highest in methanol and ethanol: methanol solvent mixture.

When compared to the DH and USP, the USB-mediated extraction yielded high color strength due to the effect of ultrasound, which promotes swelling and expansion in the pores of plant cell walls; it may have expanded the mass transfer of solute elements from plant material to extraction solvent (Boonkird et al., 2008). After the cavitation bubble has deflated, the microjet may disturb plant cells, accelerating the pace at which the solvent penetrates the tissue (Paniwnyk et al., 2001). The initial release rate of dye was relatively high due to the carotenoid concentration differential between the solvent and the plant material and the ease of extraction from the seed powder.

**FIGURE 4:**
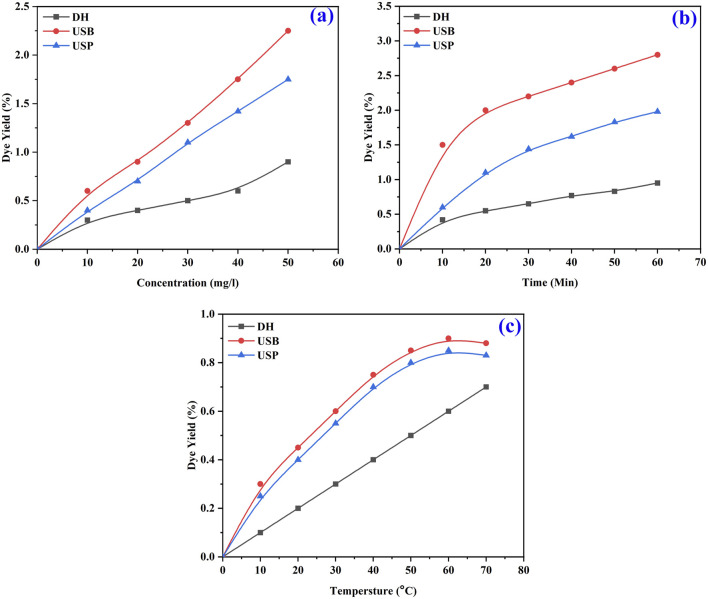
FT-IR spectra of optimized *B. orellana* dye extraction using an ultrasonic water bath and various solvents Water, methanol, ethanol, and methanol: ethanol.

The color strength of the extracted dye with the highest intensity was achieved with methanol solvent due to the interaction between the natural dye’s complex structure and the extraction solvent’s chemical characteristics (Kamel et al., 2003). Polarity changes influenced the extraction of dye from the seed powder. Based on the color strength of the extracted dye with the highest intensity, obtained using the methanol solvent and USB method, it was selected for further optimization conditions for the extraction process. Optimization of the dye extraction was studied between various temperatures, times and concentrations. Maximum dye extraction efficacy was observed at a higher concentration (0.5 g) of the seed powder and decreased with decreasing concentration in all three methods (DH, USB and USP) of extraction (Figure 4a). When the concentration increases, more seed powder is in contact with the solvent. This results in faster absorption of dye molecules. Extraction efficiency is reduced with decreasing concentration because fewer dye chemicals are available.

Similarly, a high percentage of dye yield was attained at 60 min of the extraction process (Figure 4b). The dye yield is proportional to the time spent in the extraction method, as more molecules can diffuse dye from the seed powder into the solvent. However, the yield is limited if it exceeds the threshold of diminishing returns (El-Shishtawy et al., 2003).

Further extraction time does not appreciably increase the yield since the dye chemicals present in the seed powder will eventually approach equilibrium with the solvent. Dye yield can be maximized by allowing the chemicals that make up the dye to diffuse into the solvent for 60 min. The optimum temperature for the maximum yield of dye was obtained at 60°C (Figure 4c) due to the significant influence of temperature on the extraction process. At higher temperatures, the kinetic energy of the molecules increases, promoting the diffusion of dye compounds from the seed powder into the solvent.

Fourier Transform Infrared (FTIR) spectroscopy determined the functional groups of the extracted dye. The spectral recordings covered a range of 4,000 to 500 cm^−1^ with a 4 cm^−1^ resolution. After analyzing the spectra, several distinct absorption bands were found, each corresponding to a different functional group.

**FIGURE 5:**
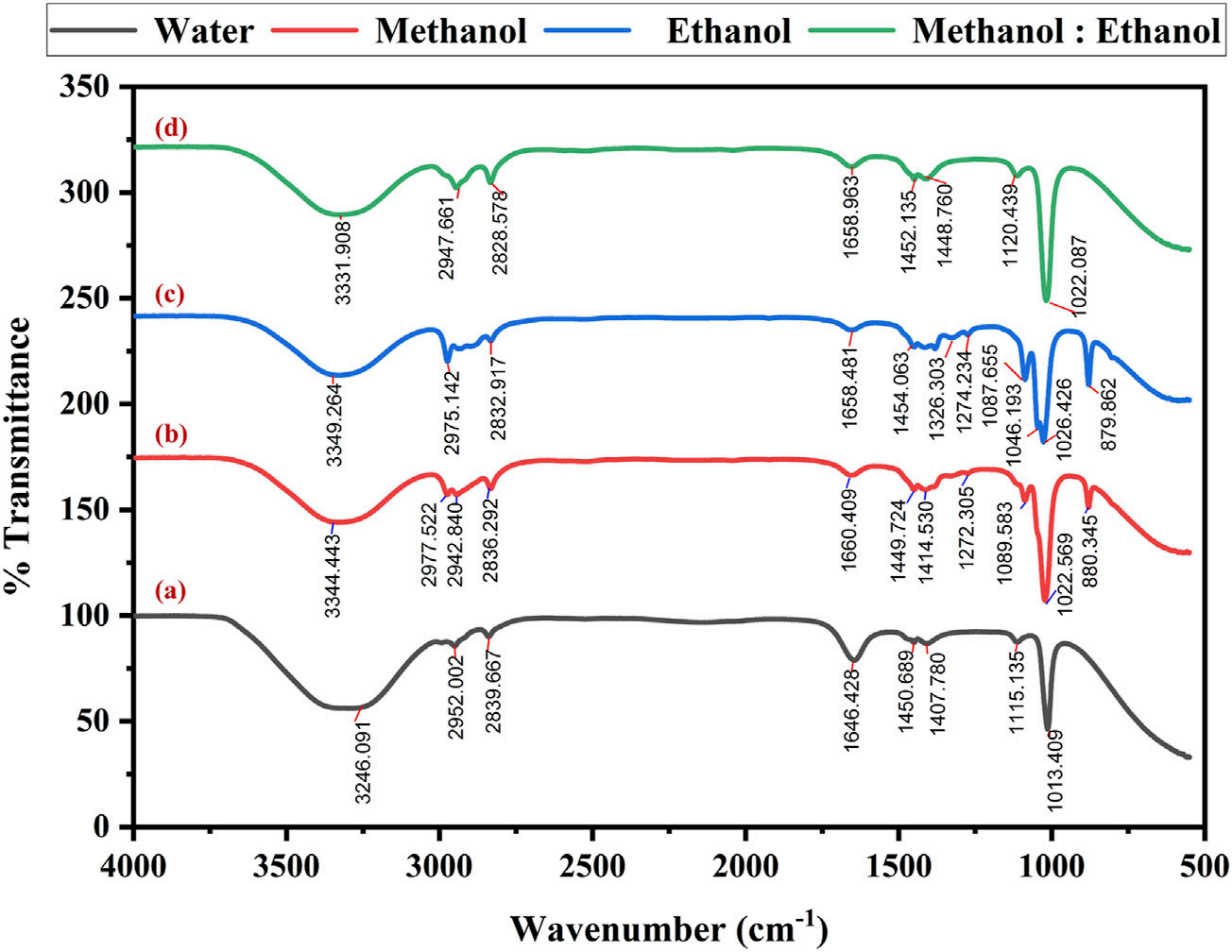
FT-IR spectra of optimized *Bixa orellana* dye extraction using an ultrasonic water bath and various solvents **(a)** Water, **(b)** methanol, **(c)** ethanol, and **(d)** methanol: ethanol.

The FTIR spectra of the dye extracted using water (Figure 5a) showed the O-H stretching alcohol group at 3,246 cm^−1^ and N-H stretching amine salt at 2,952 cm^−1^ and 2,839 cm^−1^. The band at 1,646 cm^−1^ indicate the presence of C=C stretching of cyclic alkane, and the peak value of 1,450 cm^−1^, 1,407 cm^−1^, 1,115 cm^−1^ and 1,013 cm^−1^ reveals a potent C-F stretching fluoro compound. The methanol solvent extraction of dye reveals the prominent absorption band (Figure 5b) at strong, broad O-H stretching carboxylic acid at 3,344 cm^−1^, 2,977 cm^−1^, 2,942 cm^−1^, and 2,836 cm^−1^. The band at 1,660 cm^−1^ showed medium C=C stretching alkane, followed by medium C-H alkane at 1,449 cm^−1^ and 1,414 cm^−1^. At 1,272 cm^−1^, strong C-O stretching of alkyl aryl ether was exhibited with unknown functional groups at 1,089 cm^−1^, 1,022 cm^−1^, and 880 cm^−1^, respectively.

The spectra obtained by dye extracted using ethanol reveal medium N-H stretching secondary amine at 3,349 cm^−1^ and strong O-H stretching carboxylic acid at 2,975 cm^−1^ and 2,832 cm^−1^. The peak obtained at 1,658 cm^−1^ indicates the presence of C=C stretching of alkene, O-H bending alcohol at 1,454 cm^−1^, S=O stretching sulfate at 1,326 cm^−1^, strong C=F stretching at 1,274 cm^−1^, 1,087 cm^−1^, 1,046 cm^−1^ and 1,023 cm^−1^ followed by unknown compounds at 879 cm^−1^ consistently (Figure 5c). The FT-IR spectra of methanol and ethanol (1:1) mixed solvents exhibited the peak at 3,331 cm^−1^, revealing N-H stretching primary aliphatic amine. The band specifies the presence of C-H stretching alkane at 2,947 cm^−1^, C-H stretching aldehyde at 2,828 cm^−1^, C=N stretching oxime at 1,658 cm^−1^ and C-F stretching fluoro compound at 1,452 cm^−1^, 1,448 cm^−1^, 1,120 cm^−1^, 1,022 cm^−1^, correspondingly (Figure 5d). The detected functional groups provide essential information about the extracted dye molecular structure and chemical composition.

**FIGURE 6:**
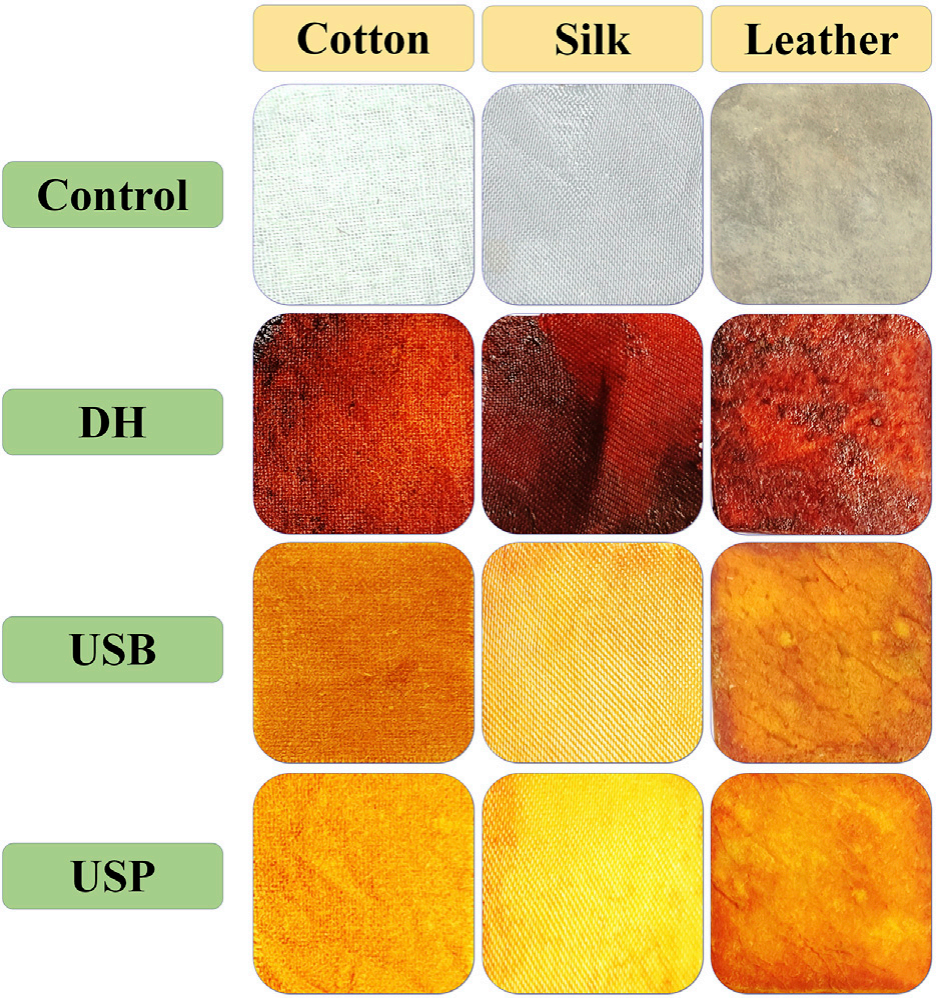
Influence of direct heating, ultrasonic water bath and ultrasonic probe of *Bixa orellana* dye exhaustion during the cotton, silk and leather dyeing process.

Similar to dye extraction, the dyeing efficiency of the extracted dye on the cotton, silk fabrics and leather was examined using three distinct methods: DH, USB and USP. The transparent color, Integ value, and colourimetric parameters of dyed cotton, silk, and leather under various dyeing methods are presented in Figure 6. Samples 1–4 exhibited apparent hues of orange, red-brown, brown, and dark brown, respectively. The sequence of samples about the Integ value was 1, 4, 3, and 2. During the process of dying in direct heating, it was observed that the color of the dye underwent a transformation from yellow-orange to brown in cotton fabric, a light orange shade in silk, and a dark brown hue in leather, as depicted in Figure 6. This phenomenon can be attributed to the oxidation of dye molecules within the dye bath, rendering them unable to withstand direct heating and reduced heating results in decreased dye absorption. The ultrasonic probe dyeing techniques have been observed to result in the deterioration of fabrics and leather when subjected to prolonged exposure. Conversely, inadequate exposure time has been found to yield suboptimal dyeing outcomes. The uniform ultrasound entering the liquor bath was effectively controlled by the water in the bath, resulting in successful ultrasonic bath dyeing without any alteration to the dye color, as depicted in Figure 6.

**FIGURE 7:**
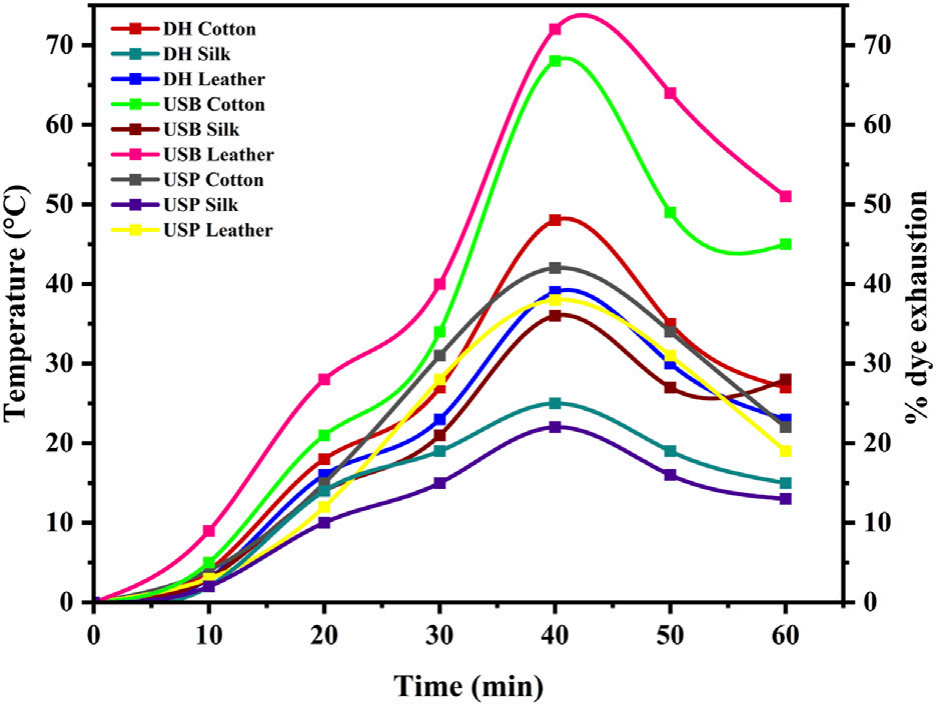
Shows the percentage of dye exhaustion in different dyeing techniques, such as direct heating, ultrasonic water bath, and ultrasonic probe, during dyeing.

Due to this occurrence, the fabric and leather consistently absorb the dye, making the dyeing process smoother without needing a moderator. (Toma et al., 2001). The outcomes of the color fastness evaluations for three distinct techniques of dyeing fabric and leather under ideal circumstances are displayed in Table 1. The L^*^, a^*^, and b^*^ values of the dyed material under the DH method were determined for cotton at 46.57, 43.13, and 51.64; for silk, were 51.95, 50.52, 59.35 and for leather, 46.65, 52.35, 51.65 respectively. Likewise, the USB method of dyed material shows the L^*^, a^*^, and b^*^ values as 80.95, 4.52, 75.35 for cotton, 88.65, −1.35, 62.85 for silk and 79.55, 015.35, 66.45 for leather accordingly. In the USP method of dyeing, the L^*^, a^*^, and b^*^ values exhibited by cotton as 88.65, −1.35, 76.65, silk as 96.45, −7.85, 53.75 and for leather as 40.32, 16.54, 68.25 consequently. Following two rounds of washing with boiling soap solution, the cotton fabric that had been dyed exhibited favourable resistance to washing and rubbing (Table 2). Figure 7 shows the dye exhaustion in different dyeing techniques like direct heating, ultrasonic water bath, and ultrasonic probe and the ratio of liquor to fabric (L: F). The optimal dyeing parameters were determined to be a temperature of 60°C, a duration of 40 min, and a liquor-to-fabric ratio of 1:10.

**TABLE 1:** Color characteristics of dyed cotton, silk fabric and leather in different dyeing methods.

Color coordination					
	L*	a*	b*	c*	h°
Direct heating					
Cotton	46.57	43.13	51.64	25.14	20.46
Silk	51.95	50.52	59.35	10.54	20.5
Leather	46.65	52.35	51.65	11.34	14.45
Ultrasonic water bath					
Cotton	80.95	4.52	75.35	10.54	48.5
Silk	88.65	−1.35	62.85	20.58	49.85
Leather	79.55	15.35	66.45	23.84	40.65
Ultrasonic probe					
Cotton	88.65	−1.35	76.65	11.34	51.45
Silk	96.45	−7.85	53.75	18.64	55.64
Leather	40.32	16.54	68.25	15.38	40.43

**TABLE 2:** Fastness property of dyed cotton, silk fabric and leather in different dyeing methods at optimum condition.

	Rubbing fastness (grade)		Washing fastness (grade)	Light fastness (grade)
	Dry	Wet		
Ultrasonic water bath				
Cotton	4–5	4–5	4	2
Silk	4	4–5	4–5	3
Leather	3	3	3–4	2
Ultrasonic probe				
Cotton	4	4	3–4	2
Silk	3–4	3–4	4	3
Leather	3	3	4–5	2
Conventional heating				
Cotton	4–5	4	4	2
Silk	4	4	3–4	3
Leather	3	3	4	2

**FIGURE 8:**
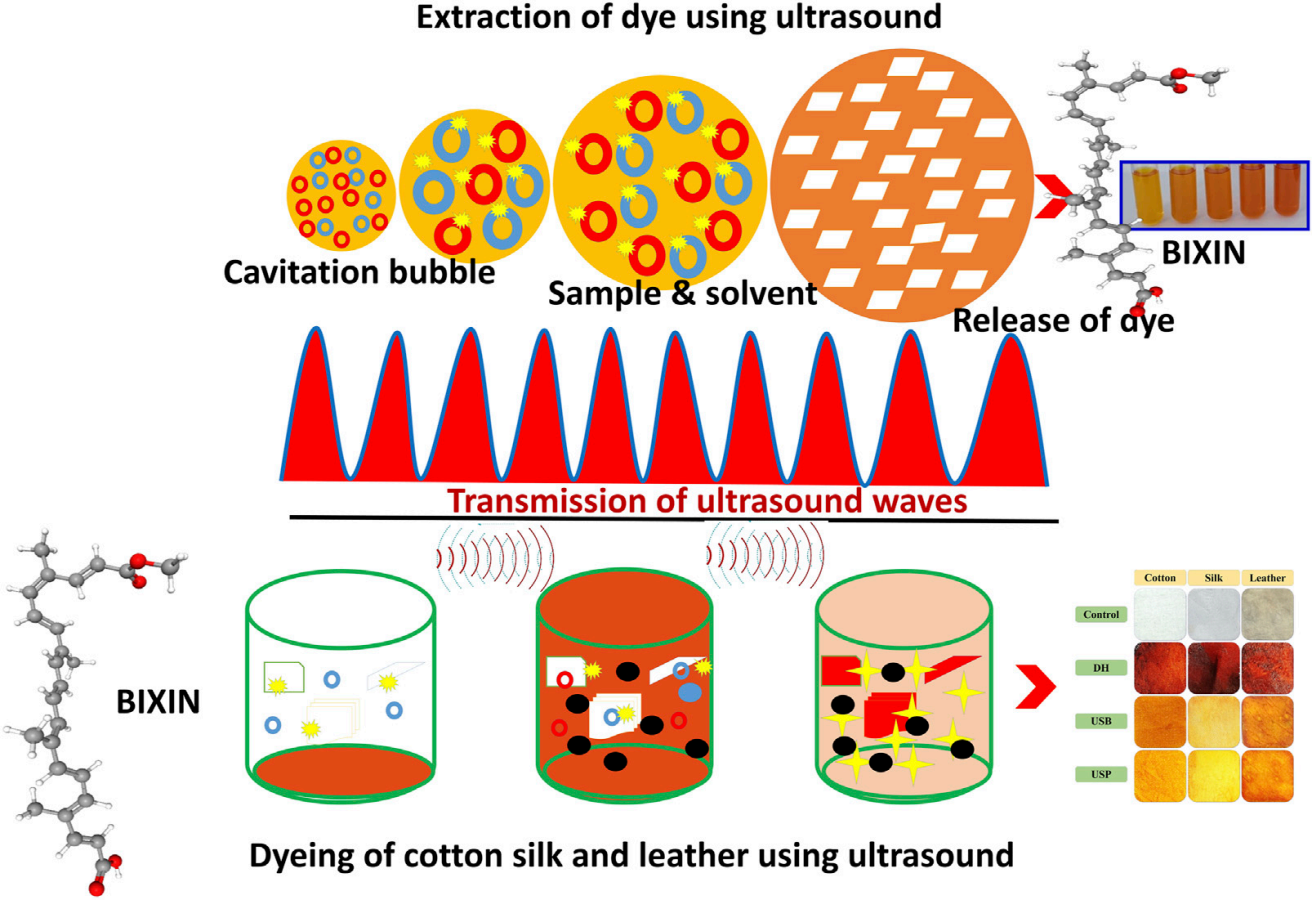
Dyeing mechanism.

The results revealed differing degrees of dye depletion in the various materials. Silk has a greater dye depletion rate than cotton and leather. Cotton had a higher affinity for the dye and absorbed more from the bath. Silk also demonstrated severe dye fatigue but slightly less than cotton. On the other hand, Leather demonstrated relatively higher dye exhaustion, suggesting a lower affinity for the dye or a different dye uptake mechanism than the other materials (Figure 8). Exploiting natural dyes to impart antibacterial characteristics to textiles and garments utilized in medical and hospital settings and preparing clothing for infants and individuals with chemical dye allergies presents a stimulating challenge in practical application. The present investigation involved the assessment of the antibacterial efficacy of dyed samples against human skin pathogens. The results indicated varying antibacterial efficacy among the dyed samples, contingent upon the textile composition and the specific bacterial strain being tested (Table 3). Overall, *Staphylococcus* sp. Exhibited the highest level of inhibition, with a reduction percentage of 85.25%, followed by *Vibrio* sp. (76.69%), *Pseudomonas* sp. (75.83%), *Klebsiella* sp. (74.24%) and *Micrococcus* sp. (74.21%) in the silk material. The reduction percentage of bacteria in cotton material revealed maximum inhibition against *Vibrio* sp. (64.79%), followed by *Staphylococcus* sp. (64.64%), *Micrococcus* sp. (62.31%), and *Pseudomonas* sp. (45.63%) respectively. *S. aureus* followed closely behind, with values ranging from 15% to 97%. Likewise, dyed leather exhibits a reduction of *Pseudomonas* sp. (66.29%), followed by *Staphylococcus* sp. (64.78%) and *Micrococcus* sp. (59.70%), *Klebsiella* sp. (55.28%) and *Vibrio* sp. (38.10%). The antibacterial properties of bixin can be attributed to a variety of mechanism strategies. Bixin has been found to induce the development of autolysins in bacteria cells, which could suppress its division and metabolic activity. This occurrence leads to the eventual breakdown and demise of the cells, as reported by Danevcˇicˇ et al. (2016), Ren et al. (2017), and Metwally et al. (2017). Moreover, Handayani et al. (2021) reported that bixin could traverse the cellular barrier by crossing the outer membrane. It also impedes the activity of certain enzymes, namely, topoisomerase IV and DNA gyrase, thereby impeding cellular proliferation.

**TABLE 3:** Antibacterial activity of the dyed material against skin pathogens.

S. No	Bacterial pathogens	Cotton			Silk			Leather		
		Undyed	Dyed	%^a^	Undyed	Dyed	%^a^	Undyed	Dyed	%^a^
1	*Staphylococcus* sp.	478	169	64.64	610	90	85.25	903	318	64.78
2	*Pseudomonas* sp.	572	311	45.63	629	152	75.83	979	330	66.29
3	*Vibrio* sp.	514	181	64.79	472	110	76.69	987	611	38.10
4	*Klebsiella* sp.	614	307	50.00	594	153	74.24	1,051	470	55.28
5	*Micrococcus* sp.	666	251	62.31	667	172	74.21	995	401	59.70

^a^ Percentage of Bacterial Reduction.

The chemical composition of cotton, a natural cellulose polymer, forms covalent bonds with natural dyes through hydroxyl groups, allowing for better dye fixation and potential antibacterial action. The larger surface area and higher porosity of cotton allow for more dye molecules to be absorbed, potentially enhancing the antibacterial effect as more dye is present to interact with bacterial cells (Emam, 2019; Ma et al., 2023). Silk, a protein-based material, contains amino acids that form hydrogen bonds or ionic interactions with dye molecules. Still, the dye uptake may vary due to its smoother surface than cotton. Silk lower porosity may limit dye uptake, resulting in less exposure of microbes to the dye, which could reduce its antibacterial efficacy (Kaur et a., 2014; Wang et al., 2024). Leather, primarily made of collagen, also forms hydrogen and covalent bonds but has additional functional groups (e.g., amines and carboxyls) that may interact differently with the dye, possibly influencing the release of antimicrobial agents. Leather, composed primarily of collagen, has a different dye absorption mechanism based on its fibrous protein matrix, which may lead to distinct bonding interactions. Depending on the tanning and finishing processes, Leather variable porosity may result in varied dye retention, influencing its antibacterial properties (Sivakumar et al., 2016; Mohan et al., 2019).

The ultraviolet protection factor (UPF) of cotton, silk, and leather dyed with *B. orellana* seed dye at its best degree of UV radiation protection indicates how effectively the dye prevents UV rays; higher UPF values are associated with more excellent protection from UV levels. The results in Table 4 demonstrate that dying fabrics made of cotton and tanned leather with *B. orellana* seed dye enhance the effectiveness of UV protection. The increase in UPF ratings and the corresponding decrease in UV transmission corroborated this finding. Cotton fabrics can receive UV protection from natural dyes, including those made from plants like *B. orellana*, due to several essential characteristics of the chemical composition of the dye and its interaction with the fabric. Bioactive compounds known to absorb ultraviolet (UV) light include flavonoids, tannins, anthocyanins, and carotenoids, which are frequently found in natural colours. *B. orellana* seed dye has carotenoid pigments called bixin and norbixin, which absorb UV radiation well. When applied to cotton fabric, these substances function as a shield, lowering the quantity of UV light that enters the material by partially absorbing or blocking it (Bonet-Aracil et al., 2016; Li et al., 2023). By forming a link with the cotton fibres, the dye molecules create an extra layer that shields the skin from UV radiation—the UV protection increases with dye absorption concentration and homogeneity. They may form strong connections because natural colors and cotton fibres include hydroxyl, amino, and carboxyl groups. The bonding improves the fabric UV absorption capacity. Natural dyes may occasionally cause a fabric’s porosity to decrease, adding barrier and reducing the materials permeability to UV light. Natural darker hue dyes offer superior UV protection (Pisitsak et al., 2016).

**TABLE 4:** Ultraviolet protection factors (UPF) for cotton, silk and leather samples dyed using *Bixa orellana* seed dye measured according to AATCC 183 (Dyed at optimum dyeing conditions).

UV protection properties	Samples (dyed at optimum dyeing conditions)					
	Cotton fabric	Control (undyed)	Silk fabric	Control (undyed)	Leather	Control (undyed)
UPF						
Dry	35.42	66.32	22.32	30.4	194.32	164.32
Wet	30.34	83.93	16.34	25.2	251.04	186.41
UV-A transmittance (%)						
Dry	25.22	48.31	22.34	16.00	124.38	110.34
Wet	29.40	58.64	30.93	12.96	146.73	120.46
UV-B transmittance (%)						
Dry	1.69	0.23	1.21	0.65	12.42	6.61
Wet	1.92	0.50	1.99	0.36	19.90	8.33

## 4 Conclusion

In conclusion, the utilization of *B. orellana* seeds for the extraction of a yellow-orange dye has shown promising results. Different solvents and extraction methods were employed, and it was determined that methanol extraction produced the most intense color across all extraction procedures. The optimized conditions for extraction involved a seed concentration of 5 g, a temperature of 60°C, and 60 min. The extracted dye was further characterized using FTIR analysis, which revealed the presence of specific functional groups indicative of its molecular structure and chemical composition. Furthermore, the dyed materials, including cotton, silk, and leather, exhibited excellent coloration using the ultrasonic water bath at 60°C for 40 min. Notably, *Staphylococcus* sp. showed the highest level of inhibition, with a reduction percentage of 85.25%. Overall, the findings of this study highlight the potential of *B. orellana* seed dye as a valuable natural resource for various applications. Its vibrant color and antimicrobial properties make it a promising candidate for use in textiles, coatings, and other industries. Further exploration and research can uncover its full potential and pave the way for sustainable and eco-friendly alternatives in the dyeing and antimicrobial fields.

## Data Availability

The raw data supporting the conclusion of this article will be made available by the authors, without undue reservation.
